# Apoptosis and autophagy markers predict survival in neoadjuvant treated oesophageal adenocarcinoma patients

**DOI:** 10.1186/s12885-022-09981-8

**Published:** 2022-08-20

**Authors:** Shereen El Mashed, Tracey R. O’Donovan, Elaine Kay, Anthony O’Grady, Damian McManus, Richard C. Turkington, Sharon L. McKenna

**Affiliations:** 1 Department of Histopathology, National Liver Institute in Egypt, Menoufia University, Shibin el Kom, Egypt; 2 Cancer Research UCC, Western Gateway Building, University College Cork, Cork, Ireland; 3 Department of Histopathology, Beaumont Hospital/Royal College of Surgeons in Ireland, Dublin, Ireland; 4 Department of Histopathology, Blackrock Clinic, Dublin, Ireland; 5 Department of Pathology, Belfast City Hospital, Belfast, UK; 6 Patrick G Johnston Centre for Cancer Research, Queen’s University Belfast, Belfast, UK

**Keywords:** Caspase-3, autophagy, LC3B, oesophageal adenocarcinoma, predictive biomarkers

## Abstract

**Background:**

Less than 20 % of patients with resectable oesophageal adenocarcinoma obtain a pathological response following neoadjuvant chemotherapy. Studies using oesophageal cancer cell lines have shown that drug sensitive tumour cells undergo apoptosis in response to drug treatment, whereas resistant cells induce autophagy and can recover following withdrawal of drug. In this study, we evaluated markers of apoptosis (active/cleaved caspase-3) and autophagy (LC3B) to establish whether these markers are useful prognostic indicators following neoadjuvant therapy.

**Methods:**

Oesophageal adenocarcinoma tumour tissue from the Northern Ireland Biobank at Queens University Belfast was examined retrospectively. Tumours from 144 patients treated with platinum-based neoadjuvant chemotherapy followed by surgical resection were assembled into tissue microarrays prior to immunohistochemical analysis. Kaplan-Meier survival curves and log-rank tests were used to assess the impact of cleaved caspase-3 and LC3B expression on survival. Cox regression was used to examine association with clinical risk factors.

**Results:**

High levels of cleaved caspase-3 were found in 14.6 % of patients and this correlated with a significantly better overall survival (*p* = 0.03). 38.9 % of patients had high cytoplasmic LC3B expression, which correlated with poor overall survival (*p* = 0.041). In addition, a distinct globular pattern of LC3B expression was identified in 40.3 % of patients and was also predictive of overall survival (*p* < 0.001). LC3B globular structures are also associated with tumour recurrence (*p* = 0.014).

When these markers were assessed in combination, it was found that patients who showed low/negative cleaved caspase-3 staining and high/positive staining for both patterns of LC3B had the worst overall survival (*p* < 0.001). Multi-variate analysis also indicated that this marker combination was an independent predictor of poor prognosis (*p* = 0.008; HR = 0.046, 95% CI = (0.005-0.443).

**Conclusions:**

The expression of cleaved caspase-3 and specific LC3B staining patterns are associated with overall survival following neoadjuvant treatment. The combination of these markers is an independent indicator of outcome in neoadjuvant chemotherapy treated oesophageal adenocarcinoma.

**Supplementary Information:**

The online version contains supplementary material available at 10.1186/s12885-022-09981-8.

## Background

The last two decades have seen a significant increase in the incidence of cancer of the oesophagus, with it becoming the seventh leading cause of cancer death in the western world [1]. There are two main histological types of oesophageal cancer, squamous cell carcinoma and adenocarcinoma; the increasing incidence is predominantly in oesophageal adenocarcinoma.

The principal treatment regimen for localized oesophageal adenocarcinoma in Europe is pre-operative platinum-based chemotherapy (neoadjuvant) or chemoradiotherapy, followed by surgical resection [2–5]. More recently the FLOT4 regimen (fluorouracil, leucovorin, oxaliplatin and docetaxel) has been demonstrated to be the most effective as a neoadjuvant therapy [6].

European 1 and 5 year survival rates for oesophageal cancer are 40 % and 12 % respectively (EUROCARE-5) [7]. This is a 3 % increase in 5-year survival since the previous 2012 EUROCARE-4 study. Recent improvements in treatment should improve these statistics in subsequent EUROCARE-6 data. However late presentation remains a problem, with many patients ineligible for surgery and drug resistance is common. Pathological classifications of tumour grade, differentiation, vascular invasion and lymph node status are sub-optimal in predicting response to neoadjuvant therapy [8]. Even following complete pathological response, there is a significant risk of disease recurrence and cancer-specific death following resection [9]. There are no specific biomarkers to guide treatment, which many patients endure with limited benefit.

Resistance to chemotherapy has been associated with decreased levels of tumour cell apoptosis. This may be due to an imbalance between the pro- and anti-apoptotic proteins of the Bcl-2 family, inability to activate caspases and / or impaired death receptor signalling. Loss of apoptosis competency can also be fundamental to carcinogenesis [10]. More recently, chemoresistance has been associated with the induction of autophagy, which promotes survival under several stressful conditions such as chemotherapy, metabolic stress and hypoxia [11–16]. Inhibition of autophagy has been reported to chemosensitise several malignancies including, CML [15, 17], ovarian cancer [18], breast cancer [19], oesophageal cancer [20] and malignant glioma [21, 22].

Although apoptosis and autophagy are functionally distinct processes, several levels of interaction have been identified. Bcl-2 family members can play a dual role in regulating apoptosis and autophagy [23]. Other tumour suppressor genes and oncogenes facilitate interaction between apoptosis and autophagy pathways and so it is imperative to select the most definitive markers for each process and simultaneously analyse both mechanisms [24].

We have previously shown that loss of apoptosis and induction of autophagy plays a major role in the resistance and recovery of chemotherapy treated oesophageal cancer cell lines. We have therefore conducted studies to evaluate relevance in patients. In a previous study, we analysed LC3B staining patterns in oesophageal adenocarcinoma tissue. A distinctive globular staining pattern of LC3B was identified as a novel prognostic marker for resectable oesophageal adenocarcinoma [25]. In this study, we examine the value of active/cleaved caspase-3 (CC3) as an apoptosis indicator and two staining patterns of LC3B, as a reflection of autophagy in a chemotherapy-treated cohort. We have assessed whether the expression levels of these markers are associated with response to neoadjuvant chemotherapy and are predictive of overall survival.

## Methods

### Patients

This study was performed and reported according to the REMARK guidelines [26]. Oesophageal adenocarcinoma tumour tissue was obtained from the Northern Ireland Biobank at Queens University Belfast. (http://www.nibiobank.org/) (https://openbioresources.metajnl.com/articles/10.5334/ojb.47/). One hundred and forty-four patients who were diagnosed with oesophageal adenocarcinoma and received platinum-based neoadjuvant chemotherapy followed by surgical resection were identified retrospectively. One core per patient was taken from paraffin embedded tumour blocks from surgical resection specimens post treatment and assembled into tissue microarrays (TMAs) at the NI Biobank. Clinical and histopathological data from this patient cohort is shown in Table 1. Chemotherapy was either ECF (epirubicin, cisplatin, 5-flurouracil) or ECX (epirubicin, cisplatin, oral 5-flurouracil/capecitabine). Two patients had carboplatin instead of cisplatin (ECarboX). 140 patients completed three cycles of chemotherapy. Four patients had two cycles. In accordance with REMARK guidelines, univariate analysis of this cohort is shown in Additional file 1.

**Table 1:** Clinical and histopathological data from oesophageal adenocarcinoma patients

Characteristics	Patient Numbers
**Gender**	
Male	115 (79.9%)
Female	29 (20.1%)
**Age**	
< 60 years old	41 (28.4%)
60-69 years old	70 (48.6%)
≥70 years old	33 (22.9%)
**Differentiation (*n*=2 unknown)**	
Well	6 (4.2%)
Moderate	53 (37.3%)
Poor	83 (58.5%)
**Tumour T histological staging**	
T I	17 (11.8%)
T II	27 (18.8%)
T III	95 (66%)
T IV	5 (3.4%)
**Tumour T clinical staging (*n*=12 unknown)**	
T I	3 (2.1%)
T II	11 (7.6%)
T III	116 (80.6%)
T IV	2 (1.4%)
**Lymphovascular invasion**	
Negative	46 (54.8%)
Positive	38 (45.2%)
**Tumour Recurrence**	
Negative	65 (45.1%)
Positive	79 (54.9%)
**Mandard classification (*n*=8 unknown)**	
Response	10 (7%)
No response	126 (87.5%)
**Tumour N stage (*n*=31 unknown)**	
N stage 0	55 (38.2%)
N stage 1	28 (19.4%)
N stage II	30 (20.8%)

### Immunohistochemical (IHC) Analysis

Deparaffinization, antigen retrieval and IHC staining for LC3B and cleaved caspase-3 were performed on an automated platform (Bond™ III system, Leica MicroSystems™, Milton Keynes, U.K.). For LC3B (Atg8b) immunostaining, a rabbit polyclonal antibody was used (ABGENT cat# AP1802a) at 1:100 dilution. Antigen retrieval is an automated process on the Bond instrument and involves the tissue being heated for 10 minutes at 100°C in ER1 retrieval buffer (Bond^TM^ Epitope Retrieval, Leica MicroSystems™, Milton Keynes, U.K.). Cleaved caspase-3 immunostaining was performed using a rabbit monoclonal antibody (Cell Signaling Technology cat# 9664; 1:200 dilution, ER1 antigen retrieval for 10 minutes). Primary antibody binding was visualized using the Bond™ Polymer Refine Detection containing a peroxide block, post primary, polymer reagent, DAB chromogen and haematoxylin counterstain.

### Quantitation of Immunostaining

#### Active/cleaved caspase-3

The staining of cleaved caspase-3 (CC3) was scored according to the extent and intensity of staining in tumour cells or tumour bed. Six high power (400x) fields per case were examined and extent of staining was scored as follows: 0 = less than 5 %, 1 = 5-25 %, 2 = 26-50 %, 3 => 50 %. The intensity of staining was scored as follows: 0 = no staining, 1 = mild staining, 2 = moderate staining, 3 = strong staining. The final score = ‘extent’ x ‘intensity’ and ranged from 0-9. In this study we defined a final score of 4 or more as positive and less than 4 as negative.

#### LC3B

Selective identification of the LC3B isotype was confirmed in our previous study by western blot analysis with recombinant LC3A and LC3B proteins [25]. In that previous study, which contained predominantly untreated patients, we scored three distribution patterns of LC3B (cytoplasmic/crescent/globular) in both untreated (Group 1; *n* = 104) and chemoradiotherapy treated (Group 2; *n* = 48) patients. Cytoplasmic expression was mainly granular and apical in distribution and there was a correlation between this apical LC3B staining and prognosis in untreated but not in treated patients [25]. In this current cohort, all patients have been treated with chemotherapy. With this cohort we noted that the cytoplasmic distribution was not apical as it was in chemo-naïve patients but distributed throughout the cytoplasm. Two patterns were then assessed in this current patient cohort: (i) intensity and extent of cytoplasmic LC3B staining and (ii) presence of LC3B globular structures.

(i) Cytoplasmic LC3B staining:

The proportion of neoplastic cells with a cytoplasmic pattern of reactivity ranged from 5–90 % per section at 100× magnification. Tumour sections were considered positive if 70 % or more of the viable tumour cells or cells in the tumour bed showed strong cytoplasmic staining.

(ii) Globular LC3B structures:

The number of globular structures was enumerated in each section at a magnification of 400× and expressed as the mean of all counts. The number of globular structures ranged from one to six (80^th^ percentile was four) per section. The tumours were subsequently classified according to 80^th^ percentile into negative (< 80^th^ percentile) versus positive (≥ 80^th^ percentile). Counting four structures or more, in five independent fields of view, was used to classify tumours as positive, while less than four were classified as negative. In addition, receiver operating characteristic (ROC) curve analysis was performed to confirm this cut off value for positivity. All slides were viewed using a DP70 Olympus digital microscope camera at 100×, 400× and 1000× (Mason Technologies, UK). Images were captured with Olympus DP-Soft823 version 3.2 acquisition software. IHC scores were assessed independently, by two pathologists (S.E-M. & E.K., authors) who were blinded to patient clinical data. Scoring was consistent in 85 % of cases. Inconsistent scores were reassessed by both S.E-M. and E.K. to assign final score. (As described in our previous study [25]).

### Statistical analysis

Statistical analysis was carried out using SPSS software (SPSS Inc., version 19; Chicago, IL). Chi-square test was used to measure the association between qualitative variables. Fisher exact test was used for 2x2 qualitative variables where more than 25 % of the cells have an expected count of less than 5. Kaplan-Meier survival curves were used to assess impact of variables on overall survival (defined as the date of surgery to the date of death from any cause). Where appropriate, Cox regression was used to give an adjusted hazard ratio and 95 % confidence interval of the effect of the different risk factors for survival. The *p*-value was considered statistically significant when it was less than 0.05. (As described in our previous study [25]).

## Results

### Immunohistochemical analysis of cleaved caspase-3 (CC3) and LC3B expression in oesophageal adenocarcinoma patient samples

Expression of cleaved caspase-3 (CC3) and LC3B was examined in TMA cores from OAC resection specimens following neoadjuvant treatment.

#### CC3 staining

**Fig. 1:**
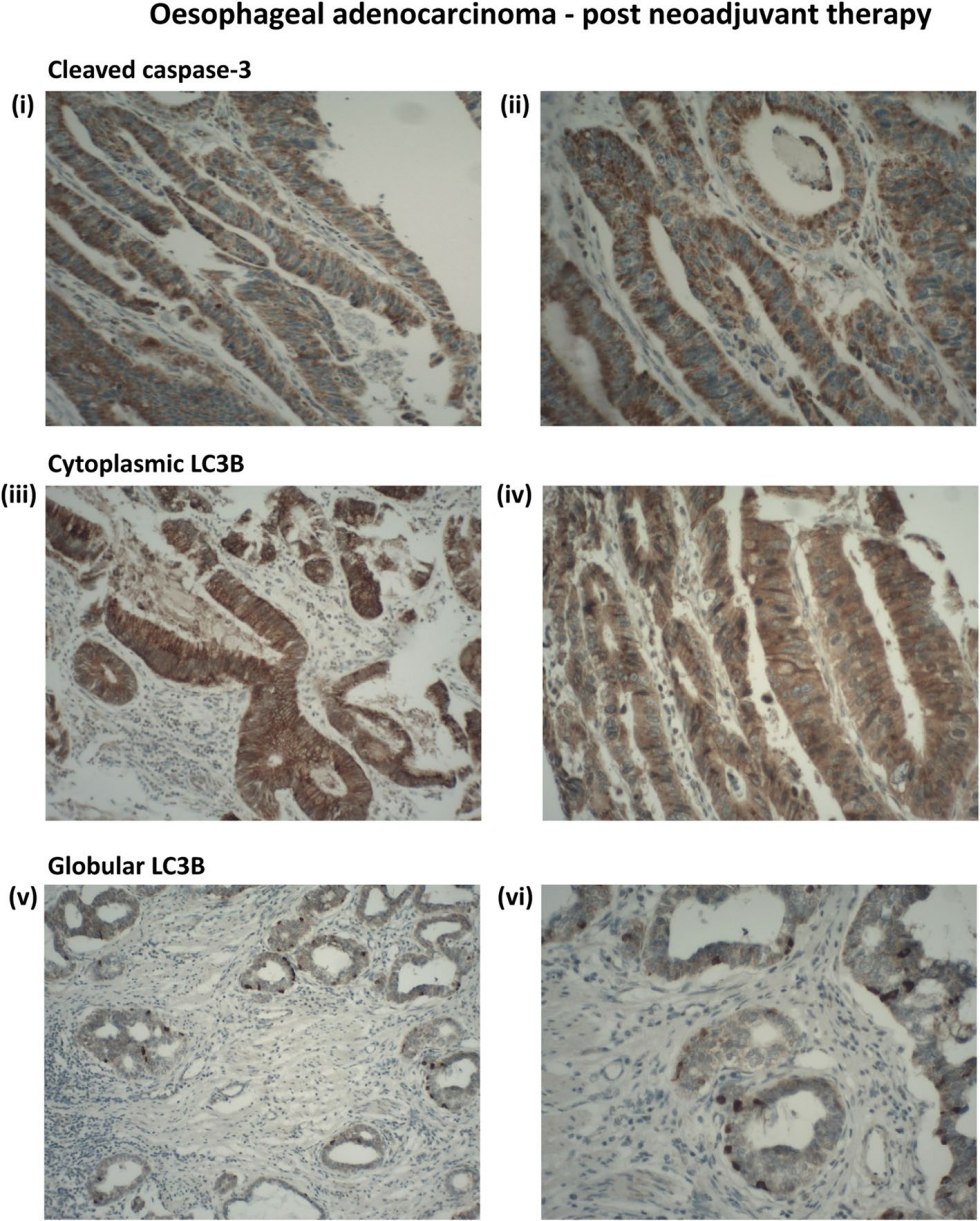
Representative positive staining for cleaved caspase-3 (CC3) and LC3B in oesophageal adenocarcinoma TMA cores from surgical resection specimens following neoadjuvant chemotherapy. Positive staining for CC3 (i) & (ii) (Magnification100x and 400x respectively). The example shown here has a score of nine. Homogenous positive cytoplasmic expression of LC3B (iii) & (iv) (Magnification100x and 400x respectively). Positive staining for LC3B globular structures (v) & (vi) (Magnification100x and 400x)

A diffuse cytoplasmic distribution of CC3 was noted in many of the sections, although the extent and intensity of staining varied. 21/144 (14.6 %) patients had positive staining for CC3. Representative positive CC3 staining in patient samples is shown in Figure 1 (i & ii).

#### LC3B cytoplasmic staining

In this current paper, we have examined a cohort of chemotherapy treated samples and observed that the distribution of LC3B cytoplasmic staining was not apical as previously reported in chemo-naïve patients [25], but was distributed throughout the cytoplasm. Tumour sections were considered positive if 70 % or more of the viable tumour cells showed strong cytoplasmic staining. Positive LC3B cytoplasmic staining was seen in 56/144 (38.9 %). Representative positive cytoplasmic LC3B staining in patient samples is shown in Figure 1 (iii & iv).

#### LC3B Globular structure

Previous work from this laboratory identified a globular pattern of LC3B staining. As these structures were found to be highly prognostic irrespective of treatment, we have incorporated this marker into the current patient cohort (all of whom are treated). In this patient cohort, 58/144 (40.3 %) of tumours displayed positive staining of globular LC3B structures. Representative images of positive LC3B globular staining pattern in these patient samples are shown in Figure 1 (v & vi).

### Correlation between apoptosis, autophagy markers and histopathological parameters

We then examined the relationship between CC3, LC3B expression patterns and clinicopathological parameters including, tumour differentiation, tumour staging, lymphovascular invasion, tumour recurrence and Mandard classification (Tables 2 and 3). Negative CC3 reactivity is associated with poor differentiation in oesophageal adenocarcinoma (*p* = 0.034). A significant association was also identified between negative CC3 reactivity and disease recurrence (*p* = 0.032) (Table 2). An association between tumour recurrence and LC3B was also identified. Positive LC3B cytoplasmic reactivity (*p* = 0.031) and presence of LC3B globular structures are associated with tumour recurrence (*p* = 0.014) (Table 3).

**Table 2:** Relationship between cleaved caspase-3 (CC3) staining and clinicopathological parameters in oesophageal adenocarcinoma patients. Statistical analysis was carried out using chi-squared test (* *p* < 0.05)

	Cleaved Caspase-3		
	Negative *n*=123	Positive *n*=21	***p***–value
**Differentiation (unknown** ***n*** **= 2)**			
Well	6 (5%)	0 (0%)	0.034*
Moderate	40 (33.1%)	13 (61.9%)	
Poor	75 (62.0%)	8 (38.1%)	
**Histological T staging**			
T I	14 (11.4%)	3 (14.3%)	
T II	20 (16.3%)	7 (33.3%)	
T III	84 (68.3%)	11 (52.4%)	
T IV	5 (4.1%)	0 (0%)	**0.216**
**Clinical T staging (unknown** ***n*** **= 12)**			
T I	3 (2.7%)	0 (0%)	
T II	8 (7.2%)	3 (14.3%)	
T III	98 (88.3%)	18 (85.7%)	
T IV	2 (1.8%)	0 (0%)	**0.568**
**Lympho-vascular invasion (unknown** ***n*** **= 2)**			
Negative	39 (32.2%)	10 (47.6%)	**0.171**
Positive	82 (67.8%)	11 (52.4%)	
**Recurrence**			
Negative	51 (61.0%)	14 (66.7%)	0.032*
Positive	72 (58.5%)	7 (33.3%)	
**Mandard (unknown** ***n*** **= 8)**			
Response *N*=10	10 (8.2%)	0 (0%)	**0.245**
No response *N*=126	107 (87.7%)	19 (90.5%)

**Table 3:** Relationship between different LC3B staining patterns and clinicopathological parameters in oesophageal adenocarcinoma patients. Statistical analysis was carried out using chi-squared test (* *p* < 0.05)

	LC3B Cytoplasmic staining			LC3B Globular staining		
	Negative ***n***=88	Positive ***n***=56	***p***–value	Negative ***n***=86	Positive ***n***=58	***p***-value
**Differentiation (unknown** ***n*** **= 2)**						
Well	5 (5.8%)	1 (1.8%)	**0.142**	5 (6%)	1 (1.7%)	
Moderate	36 (41.9%)	17 (30.4%)		33 (39.3%)	20 (34.5%)	
Poor	45 (52.3%)	38 (67.9%)		46 (54.8%)	37 (63.8%)	**0.343**
**Histological T staging**						
T I	12 (13.6%)	5 (8.9%)		12 (14%)	5 (8.6%)	
T II	19 (21.6%)	8 (14.3%)		18 (20.9%)	9 (15.5%)	
T III	55 (62.5%)	40 (71.4%)		53 (61.6%)	42 (72.4%)	
T IV	2 (2.3%)	3 (5.4%)	**0.397**	3 (3.5%)	2 (3.4%)	**0.575**
**Clinical T staging (unknown** ***n*** **= 12)**						
T I	3 (3.8%)	0 (0%)		2 (2.5%)	1 (1.9%)	
T II	8 (10.1%)	3 (5.7%)		10 (12.7%)	1 (1.9%)	
T III	67 (84.8%)	49 (92.5%)		65 (82.3%)	51 (96.2%)	
T IV	1 (1.3%)	1 (1.9%)	**0.382**	2 (2.5%)	0 (0%)	**0.089**
**Lympho-vascular invasion (unknown** ***n*** **= 2)**						
Negative	33 (38.4%)	16 (28.6%)	**0.230**	33 (39.3%)	16 (27.6%)	
Positive	53 (61.6%)	40 (71.4%)		51 (60.7%)	42 (72.4%)	**0.149**
**Recurrence**						
Negative	46 (52.3%)	19 (33.9%)	0.031*	46 (53.5%)	19 (32.8%)	
Positive	42 (47.7%)	37 (66.1%)		40 (46.5%)	39 (67.2%)	0.014*
**Mandard (unknown** ***n*** **= 8)**						
Response	7 (8%)	3 (5.5%)	**0.715**	7 (8%)	3 (5.5%)	
No response	76 (86.4%)	50 (90.9%)		76 (86.4%)	50 (90.9%)	**0.643**

### Relationship between apoptosis and autophagy markers and overall survival of patients

**Fig. 2:**
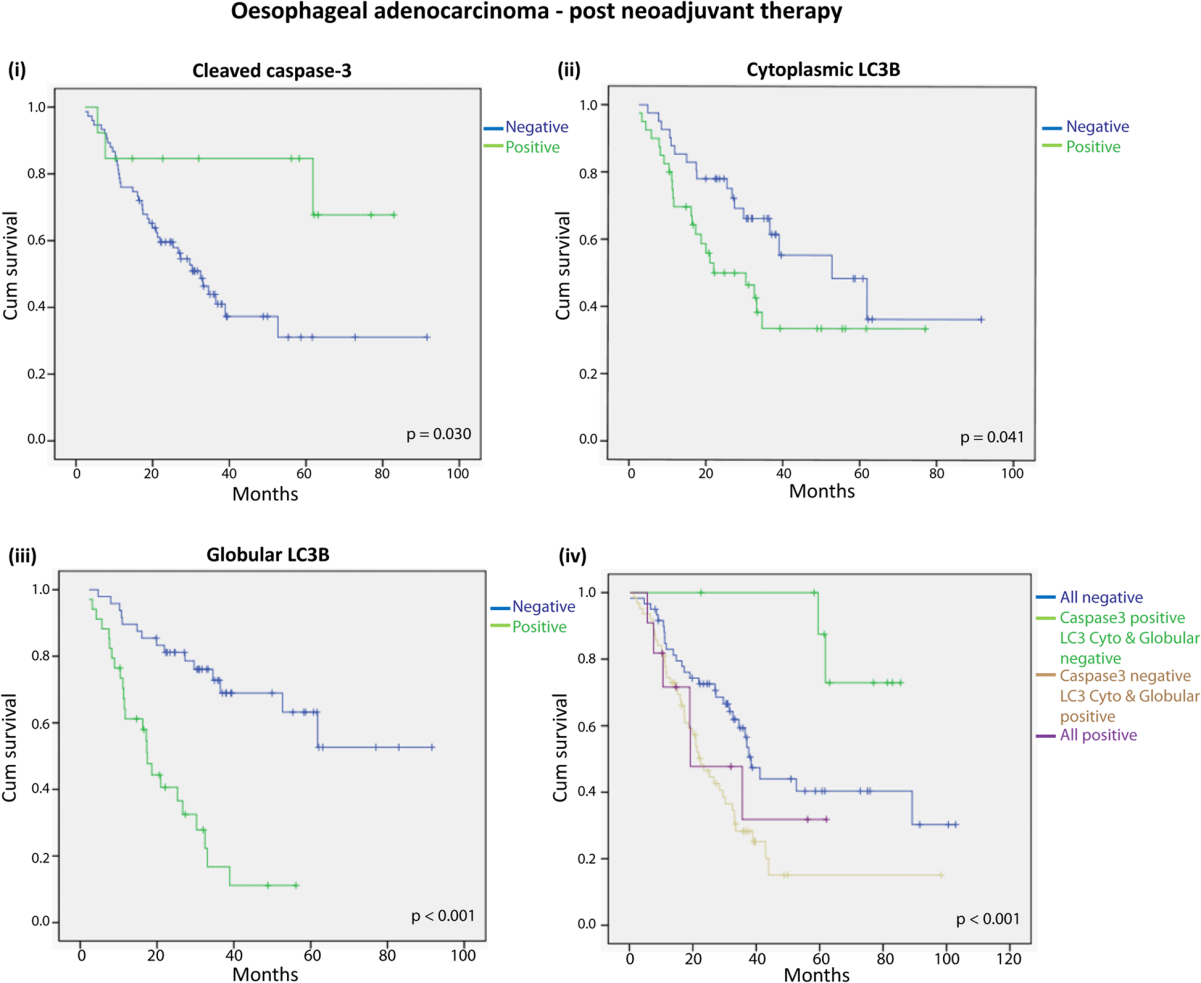
Relationship between cleaved caspase-3 (CC3), LC3B staining patterns and survival in oesophageal adenocarcinoma patients. Kaplan-Meier survival curves for neoadjuvant oesophageal adenocarcinoma patients relative to the expression of (i) CC3, (ii) cytoplasmic LC3B, (iii) globular LC3B and (iv) Kaplan-Meier survival curves for all CC3, cytoplasmic LC3B and LC3 globular structures (*p* values; Log rank test, * *p* < 0.05)

Kaplan-Meier survival curves were utilised to plot cleaved caspase-3 (CC3) or LC3B expression relative to overall survival [Figure 2]. Positive CC3 expression indicated a good prognosis with better overall survival compared with patients with negative CC3 expression (*p* = 0.030) [Figure 2(i)]. In contrast, patients who showed positive cytoplasmic LC3B staining post-treatment had a poor prognosis, with decreased overall survival compared with patients with negative cytoplasmic LC3B staining (*p* = 0.041) [Figure 2(ii)]. In addition, LC3B positive, globular structures were highly predictive of poorer outcome (*p* < 0.001) [Figure 2(iii)].

We then performed an analysis combining all markers, expression of CC3 and the two patterns of LC3B (cytoplasmic and globular structures), at each level (positive and negative). Patients who showed positive cytoplasmic CC3 staining post-treatment and negative staining for both patterns of LC3B (green curve; 5.5% of patients) had the best overall survival. In contrast, patients who showed negative cytoplasmic CC3 staining post-treatment and positive staining for both patterns of LC3B had the worst overall survival (gold curve; 30.5% of patients, *p* < 0.001) [Figure 2(iv)].

### Multivariate analysis

We then examined the independent predictive value of the marker panel. Multivariate cox regression analysis was conducted with parameters commonly associated with prognosis, including tumour differentiation, stage, lymphovascular invasion, Mandard classification, PET response, resection margins, lymph node staging and the markers panel is shown in Table 4. In the Markers panel, the third combination group in Table 4 (CC3 negative, LC3B globular and LC3B cytoplasmic positive) was an independent predictor of poor prognosis [HR = 0.046; 95% confidence interval (CI) = (0.005-0.443); *p* = 0.008]. Lymph node staging (N Stage I) was also an independent predictor of poor prognosis [HR = 0.233; 95% (CI) = (0.097-0.557); *p* < 0.001].

**Table 4:** Multivariate analysis of the panel of markers in association with other clinical pathological parameters that may have an impact on survival (* *p* < 0.05)

	Hazard ratio (95.0% CI)	***p***-value
**Differentiation**		
Mild	1(-)	0
Moderate	0.596 (0.112-3.180)	0.545
Poor	0.801 (0.46-1.394)	0.432
**Histological T staging**		
T I	1(-)	0
T II	0.938 (0.144-6.129)	0.947
T III	0.696 (0.11-4.409)	0.701
T IV	1.116 (0.219-5.688)	0.895
**Lymphovasular invasion**		
Negative v’s Positive	0.528 (0.251-1.111)	0.093
**Mandard classification**		
No response	1.586 (0.297-8.457)	0.589
Response	1.726 (0.403-7.387)	0.462
**PET response**		
PET Responder	0.845 (0.443-1.611)	0.610
PET Non-responder	0.622 (0.333-1.160)	0.135
**Markers panel**		
All are negative	1(-)	0
LC3B globular and LC3B cytoplasmic are negative and CC3 is positive	0.489 (0.165-1.446)	0.196
CC3 is negative and LC3B globular and LC3B cytoplasmic are positive	0.046 (0.005-0.443)	0.008*
All are positive	1.202 (0.565-1.658)	0.299
**Circumferential margin**		
Negative vs Positive	1.202 (0.626-2.309)	0.581
**Lymph Node stage(N stage)**		
N stage 0	1(-)	0
N stage I	0.233 (0.097-0.557)	0.001*
N stage II	0.395 (0.192-0.812)	0.012
N stage III	0.830 (0.434-1.589)	0.574

## Discussion

In this study we have shown that immuno-staining of tumour samples with an apoptosis marker (cleaved caspase-3) and an autophagy marker (LC3B) can predict outcome in neoadjuvant chemotherapy treated oesophageal adenocarcinoma. Moreover, a specific globular LC3B staining pattern is highly predictive of outcome.

Overall, levels of active/cleaved caspase-3 (CC3) and LC3B were assessed and these were found to be predictive in the setting of neoadjuvant chemotherapy followed by surgical resection. This might be expected as these tumour cells have been subjected to a stress i.e., the cytotoxic drug had been administered to the patient and their tumours responded by upregulating apoptosis or autophagy. This is analogous to previous cell line data, which would predict that an apoptotic response to treatment signifies chemosensitivity, whereas an autophagic response signifies resistance and recovery. A limitation with caspase analysis is that apoptosis is a dynamic, unsynchronized process and is difficult to capture in a snapshot of time. This is a further limitation in patient tissue, where apoptotic cells are expected to be cleared by phagocytic cells. It is possible therefore that apoptosis is underestimated in our samples. Indeed, caspase independent apoptosis may also occur. It is nevertheless remarkable, that active caspase-3 was detected after treatment, which will have ceased weeks before surgery (average 4 weeks). If apoptosis is as fast *in vivo* as it is *in vitro* [27] there must be a delayed initiation of apoptosis in some cells, following treatment. Studies in mouse models suggest that an effective response to chemotherapy requires an effective immune response [28]. We do not know what initiated apoptosis in these cells; drug effects, or a combination of drug and immune effects, but clearly the identification of active caspase-3 indicates better overall survival in this patient group.

It is important to consider that the ‘really good responders’ to treatment may influence this data, as their tissue core on the array will be comprised of tumour bed tissue. Ten patients were classed as Mandard TRG1 i.e. good responders. Caspase positive cells were not detected in these patients. It is possible that the tumour cells underwent apoptosis as part of the response to neoadjuvant therapy, but we cannot detect these cells, as they are no longer present. Caspase-3 reactivity would therefore not be of predictive value in resection specimens from TRG1 patients. Three TRG1 patients had reactivity for LC3B (Cytoplasmic or globular) in the tumour bed and this would be associated with poor prognosis overall. Again, the value of this reactivity in these patients is not clear and a much larger study would be needed to determine this. An ideal follow up study would be multi-cohort and incorporate the newer FLOT regime [6]. It would also be more useful to obtain an earlier biopsy, during the first cycle of treatment and determine if this marker pattern is predictive at an earlier stage. This could then provide an earlier opportunity to reconsider treatment options if another regime or clinical trial was available, or indeed, progress to earlier surgery.

### Other Caspase Studies

We are not aware of other studies examining the active form of caspase-3 in oesophageal adenocarcinoma. Other markers, which signify activation of caspases, such as, cleaved caspase substrates, (e.g. CK18 or PARP) may be useful biomarkers of apoptotic response. Analysis of cleaved cytokeratin18 (cCK18) has been proposed as a potential biomarker for apoptosis in carcinomas [29]. Our preliminary analysis of oesophageal cell lines found that CK18 was not always expressed (data not shown) and therefore was not further pursued in the patient samples. However, another study has analysed CK18 expression in gastro-oesophageal adenocarcinoma and found that 92 % of tumours were CK18 positive. This group compared 2 groups of patients; one had no treatment prior to surgery (primary group), while the other group was treated with platinum-based neoadjuvant chemotherapy, followed by surgery. In the neoadjuvant group, cleaved CK18 expression correlated with favourable tumour regression in univariate analysis (*p* = 0.043), but no correlation was found with multi-variate regression analysis or in survival analysis. However, surprisingly, they did find that in tumours not exposed to chemotherapy, expression of cleaved CK18 indicated improved survival. They also propose that this may reflect good susceptibility to immune mediated death [30].

### Cytoplasmic autophagy biomarker studies

Our data in this study has indicated that positive LC3B cytoplasmic staining is associated with poor survival. We have previously scored cytoplasmic LC3B staining in a different cohort of oesophageal cancer patients, from different hospitals to the current cohort [25]. In the previous study we had samples from patients who had not undergone any treatment and positive cytoplasmic LC3B staining was actually indicative of good prognosis in this group. The LC3B staining that was scored in the untreated cohort was apical – at the periphery of the cell and diffuse. A small cohort of neoadjuvant patients (n = 48) in that study was also scored in the same way and we did not see any correlation with outcome or clinicopathological parameters. This current analysis of a larger cohort has utilised a different scoring method, due to more dispersed and strong cytoplasmic staining in the treated samples and a correlation with outcome was detected. While different data sets and a smaller previous group size may also promote some disparity, clearly scoring methods and knowledge of patient treatment pre-surgery are critical for the cytoplasmic LC3B biomarker. It is possible that certain treatment regimens could induce expression or accumulation of autophagy markers more than others. In the previous study, patients also had radiotherapy as part of the neoadjuvant regime. It is also possible that different sample handling and fixing techniques could affect cytoplasmic LC3B detection and a large multi-centre sample would be required to resolve this. It is noteworthy, from both this current study and our previous one, that the globular LC3B structure is prognostic regardless of treatment Centre or whether patients had neoadjuvant therapy or not.

Another key study of oesophageal cancer has examined the prognostic value of LC3B and p62 in primary resected oesophageal adenocarcinoma in a chemo-naïve setting [31]. LC3B ‘dot like’ staining was scored as low or high and p62 was separated into 4 different (low/high) categories: (i) dot like (ii) cytoplasmic, (ii) nuclear staining and (iv) p62 sum/total. Their survival data on LC3B dot like staining is similar to our previous data on chemo-naïve patients [25] – where positive cytoplasmic reactivity to LC3B was predictive of favourable outcome. We referred to LC3B staining in this data set as apical cytoplasmic, although some of this staining would have been dot like [25], but we did not differentiate this. Adams et al, also noted apical staining, but in samples that have higher levels of dot-like staining this could be more evenly distributed [31]. Low p62 cytoplasmic staining, p62 nuclear staining and p62 sum scores all significantly correlated with a worse overall survival in their study, with a similar trend in p62 dot-like staining. When they examined combinations of markers they found that tumours with low LC3B and low p62 expression had the worst outcome (*p* = 0.005). Multi-variate analysis indicated that a combination score of dot-like/cytoplasmic p62 and nuclear p62 staining was an independent prognostic parameter (*p* = 0.033; HR = 0.6). This group did not report globular LC3B structures in their samples.

A follow up study by the same group was then conducted on 149 neoadjuvant treated oesophageal adenocarcinoma patients [32], using the same scoring methods as their 2016 study discussed above [31]. 62 patients had matched pre-and post-treatment samples. When comparing their previous treatment naïve cohort with the new neoadjuvant treated patient cohort, they noted higher overall levels of p62 dot like/cytoplasmic staining in patients who had been treated. Higher levels of p62 and LC3B dot like staining was also noted in the treated patient cohort, where they had a matched pre-treatment biopsy and post treatment samples. LC3B dot like staining did not correlate with outcome in the neoadjuvant patients. This is in contrast to our LC3B data on neoadjuvant samples (*strong (positive) cytoplasmic LC3B staining post-treatment indicated a poor prognosis*) and may be related to our different scoring method for cytoplasmic LC3B in the neoadjuvant patients. High p62 dot-like/cytoplasmic expression on its own, or in combination with low LC3B dot-like staining, was associated with poor response to chemotherapy, but there was no independent prognostic value of LC3B or p62 expression after neoadjuvant treatment [32]. As in their previous study, globular LC3B staining was not reported.

As more studies are conducted on LC3B tissue staining, the best scoring methods will undoubtedly emerge. Our study suggests that high LC3B cytoplasmic expression is associated with poor prognosis and that this might reflect a higher capacity of residual or disseminated cells to induce autophagy and recover from treatment. However, it should be noted that, some of the ‘dot like’ cytoplasmic structures are still relatively large and may represent fusion with endosomes, generating amphisomes. Their destinations (lysosomal or exosomal pathways) are also unknown and may change with tumour stage or treatment. Our own two studies suggest there is a difference in LC3B distribution when patients are treated. The functional consequence of LC3B cytoplasmic staining in tumour tissue remains to be determined.

### Globular LC3B / SLS studies

The clearest and most consistent biomarker in our patient cohorts was the presence of globular LC3B structures. These structures were always associated with poor survival. In our previous multi-variate analysis we found that the globular structure was independently associated with tumour recurrence and poor overall survival in both groups; chemo-naïve and post neoadjuvant group [25]. In this current patient cohort, LC3B globular structures did not reach significance independently. This could be related to the added number of variables in this group (5 variables in previous study, 7 in this one), or other factors such as the interval between the neoadjuvant therapy and the date of the surgery. The combination of: *CC3 negative, LC3B globular and LC3B cytoplasmic positive,* was however an independent predictor of poor prognosis.

LC3 globular structures have also been referred to as stone like structures (SLS) in other studies. LC3A globular/stone like expression patterns have been associated with tumour aggressiveness or poor prognosis in breast [33], endometrial [34], pulmonary carcinoma [35], cutaneous SCC [36], urothelial cell carcinoma (UCC) [37], colorectal cancer [38], glioblastoma [39] and gastric cancer [40] using an anti-LC3A antibody from Abgent (AP1805a). In these studies, they generally found that the diffuse cytoplasmic pattern was a poor predictor of prognosis. This antibody detects recombinant LC3A and not LC3B [41]. These SLS resemble the LC3B globular structures that we have identified as a strong prognostic marker in oesophageal adenocarcinoma.

In hepatocellular carcinoma, expression of LC3 at advanced tumour stages (but not early stages) was correlated with longer survival. Stone like structures were found to be infrequent and were not associated with prognosis. This study employed an LC3 antibody (cat# not specified) from Novus Biologicals [42]. Conversely, another group employing a rabbit polyclonal anti-LC3A antibody (Abcam) reported a ‘stone like’ pattern of LC3A expression to be an independent, highly prognostic factor in hepatocellular carcinoma [43]. Another group also identified LC3A SLS with the Abgent LC3A antibody (AP1805a) in gastric cancer. A high number of SLS was associated with increased risk of recurrence after resection of stages I-III and lower overall survival rate for stage IV [44]. A recent study on non-small cell lung carcinoma has reported that high LC3A mRNA has a significant association with poor overall survival [45]. They also reported that a higher number of LC3A stone-like structures (SLSs) was significantly associated with worse overall survival. It would be interesting to test a range of protocols on the same samples – to try and establish whether there are specific antibodies or conditions that facilitate the detection of SLS/Globular LC3A/B structures.

The biological nature and function of these globular / stone-like structures is unknown. They are clearly too large to be autophagosomes but may represent fusion of LC3B containing autophagosomes / amphisomes with a larger structure. It is possible that they represent defective autophagy or defective re-cycling. A recent study has suggested LC3 aggregates into larger structures in cells with defective autophagy [46]. It is also possible that these structures are unrelated to autophagy. A number of other cellular processes (e.g. entosis) can use autophagy proteins including LC3B (reviewed in [47]). Further molecular studies and new biomarkers of other related processes will be needed to fully characterise these SLS / globular structures and this may help in their more reliable detection and utilisation as a biomarker.

## Conclusions

This is the first combined analysis of apoptosis and autophagy markers in oesophageal adenocarcinoma. We have demonstrated an association between cleaved caspase-3 and LC3B and survival of neoadjuvant treated patients. We have previously shown that LC3B globular structures are highly predictive of patient outcome. This current study further indicates that a combination of apoptosis and autophagy markers is likely to be optimal for development of a reliable predictive test in the future. Analysis of a larger multi-centre cohort with earlier biopsies would be required to validate and optimise the utility of these markers. It is hoped that such a test could identify a high-risk group of patients that could be offered an alternate therapy, earlier surgery, or additional adjuvant therapy, depending on the availability of agents in the future, to improve their overall outcome.

## Supplementary Information

**Additional file 1.** Univariate analysis of clinical, pathological and histological data of all patients relative to survival. Data is analysed by Kaplan-Meier (Log rank) test.

## Data Availability

All data generated or analysed during this study are included in this published article and its supplementary information files.

## Abbreviations

LC3B: Microtubule-associated protein 1 light chain 3 beta (MAP 1LC3B)
CC3: Cleaved Caspase-3 (active caspase-3)
5-FU: 5-fluorouracil
TMAs: Tissue microarrays
IHC: Immunohistochemistry
DAB: 3, 3’-diaminobenzidine
CI: Confidence interval
SLS: Stone-like structures
OAC: Oesophageal adenocarcinoma
