# Supplementary material for: Apoptosis and autophagy markers predict survival in neoadjuvant treated oesophageal adenocarcinoma patients
Source: BMC Cancer. 2022 Aug 20;22:908. doi: 10.1186/s12885-022-09981-8 (PMC9392302; doi:10.1186/s12885-022-09981-8)
Supplement: Supplementary file 1 — Additional file 1. Univariate analysis of clinical, pathological and histological data of all patients relative to survival. Data is analysed by Kaplan-Meier (Log rank) test. [file 12885_2022_9981_MOESM1_ESM.pdf]

Additional file 1. Univariate analysis of clinical, pathological and histological data of all patients relative to survival. Data is analysed by Kaplan-Meier (Log rank) test.

Variables		Overall survival<br>Mean 95% Confidence Interval	SE	Log Rank test	p-value
**Tumour differentiation**	Well<br>Moderate<br>Poor	76.309(47.874-104.745)<br>55.348(44.069-66.627)<br>39.744(40.215-54.643)	14.508<br>5.755<br>4.462	10.517	0.005
**Mandard classification**	No response<br>Response<br>Unknown	48.326(40.654-55.999)<br>30.342(19.331-41.353)<br>43.181(24.136-62.226)	3.915<br>5.618<br>9.717	0.636	0.727
**Lymph node stage**	N0<br>N1<br>N2<br>N3	77.170(66.040-88.300)<br>43.812(33.343-54.280)<br>26.678(17.600-35.756)<br>17.235(13.017-21.453)	5.678<br>5.341<br>4.632<br>2.152	63.017	< 0.001
**Lymphovascular invasion**	Negative<br>Positive	73.394(60.926-85.862)<br>32.898(26.298-39.498)	6.361<br>3.367	25.393	< 0.001
**Histological T stage**	T1<br>T2<br>T3<br>T4	59.205(47.893-70.517)<br>72.094(55.864-88.324)<br>37.110(29.815-44.406)<br>20.059(8.541-8.541)	5.771<br>8.281<br>3.722<br>5.877	19.489	< 0.001
**Circumferential Surgical margin**	Negative<br>Positive	66.664(56.591-76.737)<br>24.889(20.299-29.479)	5.139<br>2.342	36.177	< 0.001
